# A continuous real-world dataset comprising wearable-based heart rate variability alongside sleep diaries

**DOI:** 10.1038/s41597-025-05801-3

**Published:** 2025-08-23

**Authors:** Aitolkyn Baigutanova, Sungkyu Park, Marios Constantinides, Sang Won Lee, Daniele Quercia, Meeyoung Cha

**Affiliations:** 1 Korea Advanced Institute of Science and Technology (KAIST), School of Computing, Daejeon, 34141 Republic of Korea; 2 Institute for Basic Science (IBS), Daejeon, 34126 Republic of Korea; 3 KDI School of Public Policy and Management, Sejong, 30149 Republic of Korea; 4 Nokia Bell Labs, Cambridge, CB3 0FA United Kingdom; 5 CYENS Centre of Excellence, Nicosia, 1016 Cyprus; 6 Kyungpook National University Chilgok Hospital, Department of Psychiatry, Daegu, 41566 Republic of Korea; 7 Politecnico di Torino, Torino, 10129 Italy; 8 Max Planck Institute for Security and Privacy (MPI-SP), Bochum, 44789 Germany

**Keywords:** Quality of life, Interdisciplinary studies

## Abstract

Wearable technology is a promising tool for everyday health monitoring, with heart rate variability (HRV) providing key insights into current and potential health conditions. However, previous HRV datasets were collected under controlled clinical conditions, rather than in complex real-world environments. Here, we collected continuous physiological and motion signals using smartwatches from 49 healthy individuals (mean age: 28.35 ± 5.87, 51% females) over four weeks. The recordings were sampled every 100 ms, allowing for short-term HRV computation based on 5-minute segments of raw data. We validated the data by examining the frequency of collected signals, analyzing the correlation between the smartwatch sensor data and computed HRV, and demonstrating the presence of HRV and sleep-related feature distributions expected from the literature. Our wearable recordings were collected alongside daily self-reported sleep diaries and biweekly clinical questionnaires that assessed anxiety, depression, and insomnia. The dataset aims to benchmark in-the-wild HRV recordings, enable future analyses in the field, and support the development of predictive analytics that use sleep patterns and wearable data as health indicators.

## Background & Summary

Given that they continuously monitor physiological parameters, wearable technologies have become increasingly popular for capturing everyday activity, motion, sleep patterns, and other health measurements. In particular, wrist-worn wearable devices have proven effective as screening tools for post-traumatic stress symptoms^1^ and in facilitating continuous monitoring of blood pressure^2^ and metabolic activity^3^. Wearables can also provide insight into mental health, an integral component of overall well-being, and there is an increasing impetus to monitor depression, anxiety, and other conditions using such devices. Specifically, metrics such as sleep duration and consistency, commonly tracked by these devices, show strong associations with self-reported mood and depression scores^4^. Additionally, sleep quality can affect weight homeostasis^5^, potentially contributing to obesity development. Furthermore, poor sleep quality is predictive of increased anxiety and depression, reduced quality of life, muscle mass, and strength in individuals with obesity^6^.

Heart rate variability (HRV) is one measurement that is widely collected by and can be extracted from wearables. Most commercial devices, such as Fitbit and Galaxy Watch, record Photoplethysmography (PPG) signals, which monitor changes in blood volume based on light absorption of reflected light by the skin. The Samsung Galaxy Watch Active 2, which was used in our study, specifically employs green LED light for PPG measurements. PPG signals can then be used to derive HRV, a biological marker that provides an indirect assessment of autonomic nervous system function since the interplay between the sympathetic and parasympathetic systems influences heart rate^7^. Reduced heart rate variability and nighttime heart rate reduction, particularly among individuals with insomnia and depression, have been linked to lower parasympathetic activity, which is potentially connected to cardiovascular risk in these populations^8,9^. Some studies have linked chronic stress to a reduction in resting-state HRV^10^, while others suggest that the nonlinearity of HRV patterns is associated with healthy aging^11^. Moreover, it has been demonstrated that symptoms of depression are associated with decreased HRV, with the association explained by the use of antidepressants^9,12^.

Continuous monitoring of HRV is thus potentially a valuable indicator of human health. However, most research to date relies on short-term (i.e., 5-minute) HRV measurements obtained from Electrocardiography (ECG)^10^ or PPG^13^ data gathered in clinical settings. Some studies employ long-term (i.e., 1 to 24 hours) HRV measurements, deriving indices over 24-hour intervals^14,15^. Yet, such one-time measurement intervals, whether taken over the long- or short-term, cannot capture the complete cycle of daily life activities. This is because factors associated with HRV, such as psychological conditions like depression and physiological states like sleep, may fluctuate over time^16^. These fluctuations make it challenging to accurately assess HRV with a single measurement. Performing continuous daily HRV assessments thus becomes essential for analyzing changes over longer periods, but progress in this area has been limited, in part by the lack of publicly available datasets of *in-situ* HRV measurements (i.e., real-world HRV measurements).

**Fig. 1:**
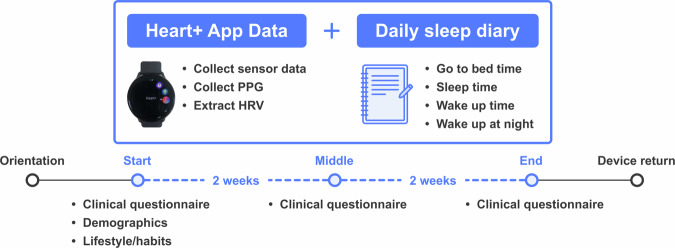
Overview of the study design over four weeks. Daily smartwatch measurements, including sensor data, PPG, and HRV, were collected through the smartwatch application, while participants recorded bedtime, sleep onset, wake-up time, and nighttime awakenings in daily sleep logs. Standardized clinical questionnaires were administered at the beginning, midpoint, and end of the study.

Recognizing the valuable health insights provided by wearable technology, our primary objective in this study was to collect and share raw and processed *in-situ* data from wearable devices, enriched with sleep logs and information on mental health conditions from the same individuals. We present a dataset comprising 49 participants (aged 21–43, including 25 females) collected over four weeks using Samsung Galaxy Active 2 smartwatches (Fig. 1). Our data allows researchers to observe measurements at three levels: daily, weekly, and monthly, broadening the potential for new analyses and providing data to guide new application development. The dataset includes continuous motion recordings with short-term HRV measurements derived from PPG signals, lifestyle data from participants, biweekly clinical questionnaires, and self-reported daily sleep diaries. While raw sensor data from such devices may be available from wearable-producing companies, our dataset is enriched by the sleep logs and psychological questionnaires. We validate the data by comparing expected results with published data and by examining observed patterns through daily-level distributions and correlations.

The dataset presented by this work offers a valuable resource for the development of applications aimed at monitoring human health and will support the exploration of associations between HRV and potential shifts in mental states, specifically depression, insomnia^17^, and anxiety. Sleep researchers may find it particularly useful to investigate how nightly sleep patterns relate to HRV indices on preceding and subsequent days. Unlike previous studies, which largely focus on specific groups such as pregnant women^13^ or individuals with certain medical conditions^9,12^, our dataset comes from average healthy individuals, providing a resource for developing predictive analytic tools.

## Methods

### Data collection setup

We chose the Samsung Galaxy Active 2 for dataset collection since, at the time of the study (2021), it was one of the few devices that (1) allowed third parties to access raw PPG data through the development and embedding of a wearable application and (2) enabled convenient adjustment of the sampling rate of features via the app. In addition, prior studies had validated the measurement accuracy of this device^18^. Data collection began with an in-person orientation session where we explained experimental details, including the purpose and duration of the study, data collection methods, participation rewards, and instructions for using the wearable device. Participants were given a smartwatch and instructed to wear it on the wrist of their non-dominant hand at all times during the experimental period, except during sleep, when they were asked to charge the device. Although the wearable device is water-resistant, participants were advised to remove it during long baths or swimming.

### Data collection

#### Procedure

Figure 1 illustrates the overall data collection workflow. The initial orientation was followed by four weeks of data gathering, concluding with device reclamation. A pre-survey was conducted at the outset to gather general demographics and lifestyle information from the participants. During the data-gathering period, participants completed a short online survey assessing their mental health three times (i.e., two-week intervals). We evaluated insomnia using the Insomnia Severity Index (ISI)^19^ questionnaire. We also surveyed mental health using PHQ-9 (Patient Health Questionnaire-9)^20^ and GAD-7 (General Anxiety Disorder-7)^21^ to assess depression and anxiety, respectively.

#### Application

We developed a wearable device application named “Heart+” to facilitate data collection. This application gathered three types of data: (1) activity, based on an accelerometer, gyroscope, gyroscope rotational vector, and pedometer; (2) physiological, including heart rate and PPG; and (3) environmental sensing, specifically recordings of ambient light. Built on the Tizen platform (https://www.tizen.org/)—Samsung’s operating system designed specifically for embedded programming—the application was configured to sample data at 100 ms intervals (i.e., 10 Hz).

PPG sampling frequencies vary widely in the literature, with studies using rates ranging from 5 Hz to 100 Hz and recent experiments sampling at 20–25 Hz^22,23^. We selected 10 Hz to balance temporal resolution against signal processing principles and practical constraints. More specifically, according to the Nyquist theorem, when sampling a continuous signal, the sampling rate should be at least twice the highest frequency to be captured. Since human heart rates range from 40–220 bpm (i.e., 0.67–3.67 Hz), a minimum sampling rate of 7.34 Hz (2 × 3.67 Hz) is theoretically sufficient to capture the maximum heart frequency^24^. Additionally, studies demonstrate that interpolation methods can enhance HRV accuracy from lower temporal resolution PPG signals, supporting our sampling frequency decision^22^.

In terms of practical considerations, adjusting sampling frequency is a key strategy for reducing computational load and power consumption due to limited system resources in wearable devices^25^. The selected sampling frequency enables continuous recording throughout the day while maintaining a battery life of up to 14 hours. Thus, 10 Hz provides an adequate margin above the theoretical minimum while minimizing battery consumption—a key consideration for uninterrupted 24/7 monitoring over four weeks. Lower sampling rates also reduce power consumption, memory, and data transmission burdens^22^. Finally, the heart rate monitor on this device, provided by the Samsung Health application developed by Tizen, uses an internal proprietary algorithm to automatically record real-time heart rate based on PPG signals. All of these signals are temporarily stored on the watch and transmitted to the server at regular intervals (every 30 minutes) when connected by Wi-Fi, thus minimizing battery use^24^.

We used the Tornado framework (https://www.tornadoweb.org/) to create a RESTful API that facilitates communication between the application and the server. This API has two endpoints: the first stores data from the registration portal and generates unique user identifiers, and the second handles the storage of all sensor data retrieved from the watch.

The data, including user ID and sign-up time, were stored in a MongoDB Instance within the Users collection. Device identifiers, such as device ID and remaining battery ratio, were stored in the Devices collection. Meanwhile, continuous raw sensor data was packaged into CSV files and locally stored by the watch until transmission to the server along with the device ID. Before storing, the server referenced the MongoDB collections, matched the user ID with the transmitted device ID data, and sorted the CSV files into directories categorized by user ID. Once the watch connected to Wi-Fi, the packaged CSV files were transferred to the server according to user IDs, and the transferred files were then deleted from the watch locally.

#### Monitoring

We monitored the data collection process to ensure it was conducted properly. First, we reviewed the recordings from wearables and sleep diaries daily. Participants were informed that the research team would monitor daily recordings solely to verify that the smartwatch was worn correctly and that the data were stored accurately. If a wearable device was not worn (1) more than three times or (2) for longer than two hours per day (excluding sleep time), an individual warning was sent. Additionally, we checked daily to ensure that all participants logged an entry in their sleep diary.

Second, we set up an online chat room through a social network messenger. Reminders to wear the watch and complete daily tasks, such as connecting to the Heart + app for data storage and filling out daily diaries, along with important announcements (e.g., the three clinical surveys), were sent via this group chat. Participants were also instructed to report any issues in the chat and were provided with an emergency contact number for urgent questions and updates.

### Ethics statement

The dataset workflow was developed with and approved by the Institutional Review Board of KAIST (KH2020-027). We obtained written informed consent from participants, using a form that outlined the purpose, duration, and procedure of data collection. All participants agreed to the use of anonymized personal information for research purposes and were compensated with USD 100 if they faithfully participated in the study to its completion. The most diligent 10 participants were rewarded with an extra coffee coupon. All data was anonymized before release, ensuring compliance with the privacy rights of the participants.

### Participant recruitment and preparation

Our objective was to collect real-world daily sensor data along with information on sleep and mental health in healthy individuals. We recruited participants from a university and a research institution in South Korea through online postings and flyers. The recruitment announcement specified the following eligibility criteria: (1) aged 20 to 50, and (2) not undergoing any hospital treatment for acute medical, surgical, or psychiatric illness. Ultimately, 49 participants were recruited for a four-week experiment.

**Fig. 2:**
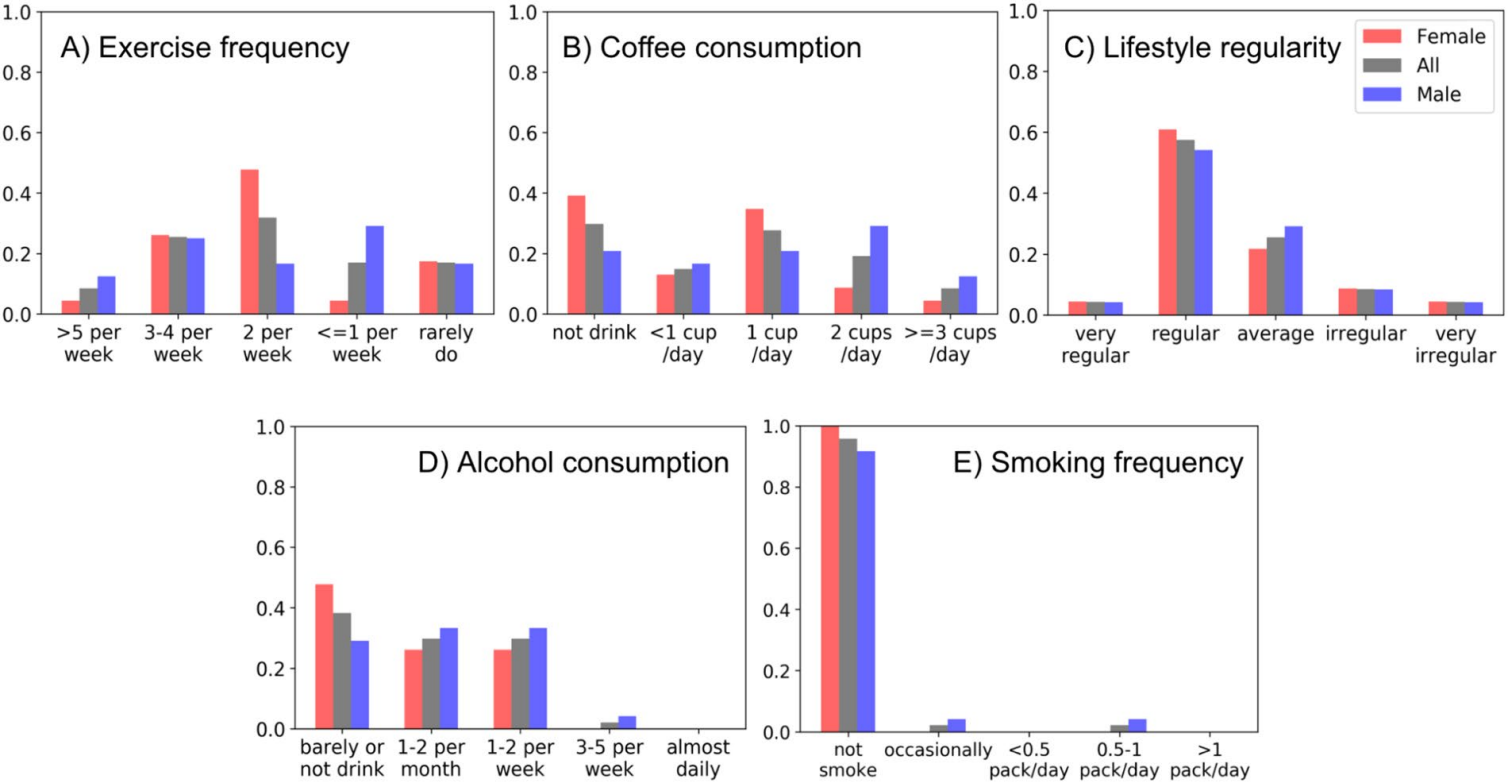
Distribution of participant lifestyle survey responses. The figure illustrates the frequency of participant responses across five categories: weekly exercise frequency (**A**), daily coffee consumption (**B**), overall lifestyle regularity (consistency in daily activities) (**C**), alcohol consumption frequency (**D**), and smoking frequency (**E**). Data are presented for all participants, with bar colors indicating female (red), male (purple), and all (grey) participants.

**Fig. 3:**
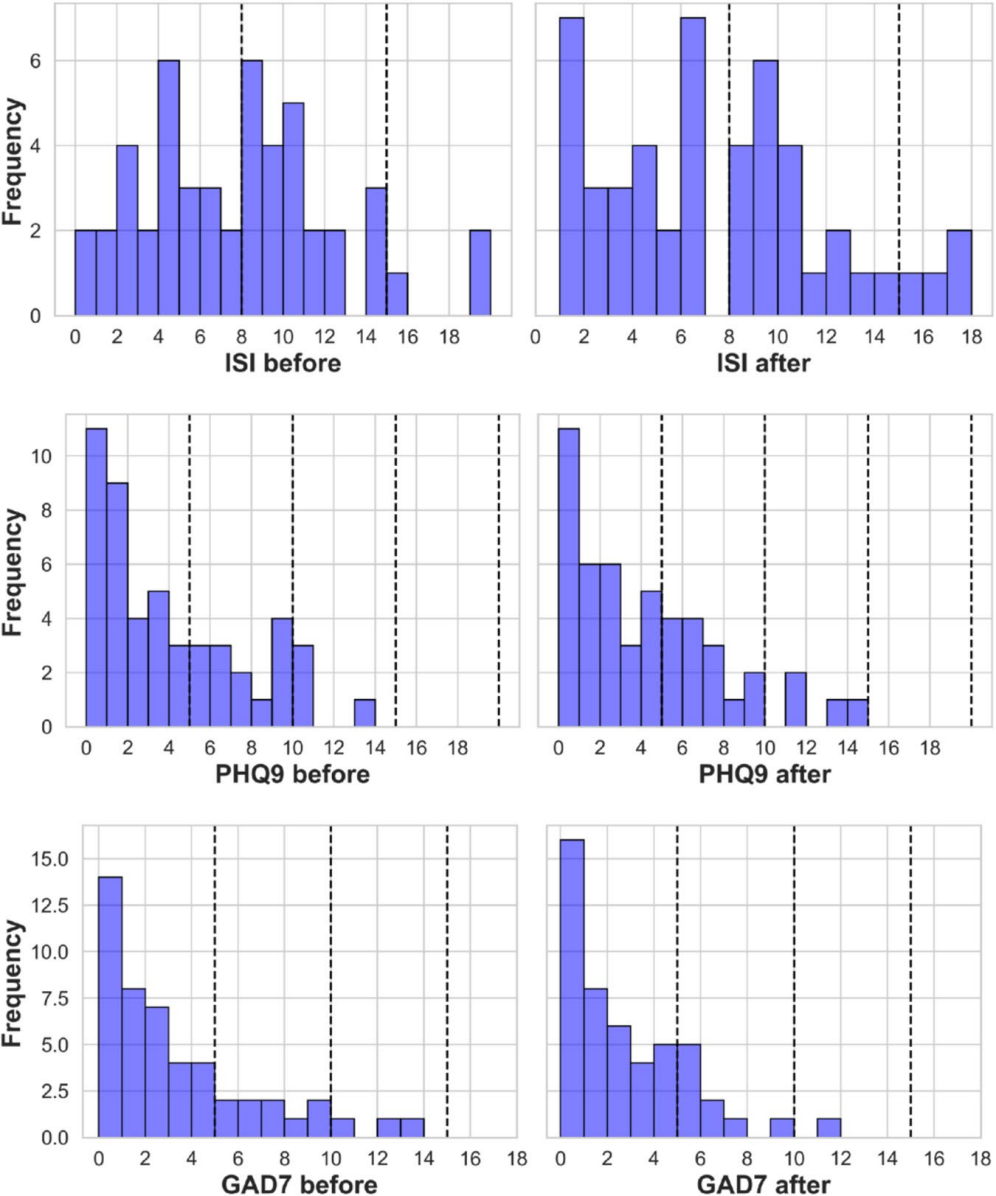
Distribution of participant scores from clinical questionnaires, including insomnia (ISI: top), depression (PHQ9: middle), and anxiety (GAD7: bottom). The charts show scores before data collection started (left panel) and 4 weeks later, after the study was completed (right panel).

Table 1 summarizes participant demographic information both in aggregate and stratified by gender. The participants were balanced across three categories, which included office workers (35%), undergraduate students (30%), and graduate students (35%). Figure 2 presents the distribution of participants by lifestyle factors such as smoking, exercise frequency, consumption of alcohol and coffee, and overall lifestyle regularity, all of which were self-reported. The vast majority of participants reported being non-smokers, consuming alcohol at most once a week, and maintaining a regular lifestyle. Participants were relatively balanced in their exercise frequency and daily coffee consumption levels. The distribution of participants based on scores from clinical questionnaires assessing insomnia (ISI), depression (PHQ9), and anxiety (GAD7) is presented in Fig. 3. Although these assessments were conducted at three time points—before, midway, and after the experiment—we report the distributions from the pre-experiment and post-experiment assessments (i.e., four weeks apart), for visual conciseness. All the scores are available in the provided data.

**Table 1:** Demographic characteristics of participants.

	All	Female	Male
**Participants**	49	25	24
**Age (years)**	28.35 ± 5.87	29.12 ± 6.03	27.54 ± 5.72
**Height (cm)**	168.80 ± 6.40	164.37 ± 4.23	173.47 ± 4.75
**Weight (kg)**	62.89 ± 11.48	54.47 ± 4.66	71.31 ± 9.97
**Marital Status**	Single: 90% Married: 8% No answer: 2%	Single: 84% Married: 12% No answer: 4%	Single: 96% Married: 4% No answer: 0%
**Occupation**	Undergraduate: 30% Graduate: 35% Office worker: 35%	Undergraduate: 32% Graduate: 16% Office worker: 52%	Undergraduate: 29% Graduate: 54% Office worker: 17%

Mean values (±standard deviations) are provided for age, height, and weight. Categorical data, including marital status and occupation, are presented as percentages.

### Data processing

The final dataset collected over the four weeks includes five types of data: (1) participant demographics, (2) smartwatch sensor data, including PPG signals, (3) computed HRV features, (4) sleep diaries, and (5) biweekly clinical survey results. The dataset also contains demographic and lifestyle information of participants matched to their device IDs. To process raw signals from wearable devices, we referred to the official Tizen documentation for sensor descriptions, filtering out values outside the acceptable range for each sensor before aggregating them into 5-minute chunks (https://docs.tizen.org/application/native/guides/location-sensors/device-sensors/). Additionally, we used sensor recordings to detect periods when smartwatches were off-wrist (e.g., not being worn) by excluding any data lacking a heartbeat frequency or heartbeat values outside the acceptable range. The research team manually inspected the sleep diaries to ensure their accuracy, correcting any AM/PM confusion by participants. Through daily monitoring, we detected issues with two participants (i.e., device IDs ab50 and kb24) during the first three days of the experiment. These participants were instructed to wear the device for an additional three days beyond the end of the study.

## Data Records

The dataset is available on Figshare^26^ and can be accessed through 10.6084/m9.figshare.28509740. The dataset includes five types of data: participant demographics and lifestyle, clinical questionnaires, raw sensor data, sleep diaries, and continuous 5-minute HRV measurements. Data was anonymized by using a device ID. The dataset consists of four CSV files—separate files containing data from the smartwatch sensors, sleep diaries, and user surveys—a subdirectory with raw wearable sensor data (*raw_data*), and a README document as described below. Table 2 provides a detailed description of the features contained in our dataset.

**Table 2:** Overview and descriptive statistics of the data measures included in the dataset.

Type	Feature		Description
Smartwatch	HR		Heart rate monitor: the user’s heart rate
	ibi		Interbeat: interval between successive heartbeats (also RR interval)
	acc		Accelerometer: change in the velocity
	grv		Gyroscope rotational vector: orientation of the device
	gyr		Gyroscope: angular velocity of the device
	steps		Pedometer: number of steps
	distance		Pedometer: moving distance
	calories		Pedometer: burned calories
	light		Light sensor: ambient light intensity
	PPG		LED green batch: amount of light reflected from skin
HRV	Time-domain	SDNN	The standard deviation of RR intervals
		SDSD	The standard deviation of the successive RR intervals
		RMSSD	Root mean square of successive RR intervals
		PNN20	Percent of successive RR intervals that differ by more than 20 ms
		PNN50	Percent of successive RR intervals that differ by more than 50 ms
	Frequency-domain	LF	Absolute power of the low-frequency band
		HF	The absolute power of the high-frequency band
		LF/HF	The ratio of LF to HF
Sleep diary	in-bed duration		Total number of minutes spent in bed
	sleep duration		Total sleep time
	WASO		Total number of minutes awake after having initially fallen asleep
	sleep efficiency		The ratio between the total sleep time to the time spent in bed
	sleep latency		Total number of minutes taken to fall asleep
Questionnaire	MEQ		Morningness Eveningness Questionnaire
	PHQ9		Presence and severity of depression
	ISI		Insomnia Severity Index
	GAD7		Anxiety Test Questionnaire

### Dataset contents

*survey.csv* includes user-reported data encompassing participants’ profiles and clinical questionnaire scores, which were collected three times. Each row represents information about one participant.

1. User profiles. For each user, the table includes their anonymized device ID, demographics (age, gender, height, weight, marital status, and occupation), and lifestyle factors (smartwatch experience, lifestyle regularity, exercise frequency, smoking and drinking frequency, and coffee consumption level).
2. Clinical questionnaires. Participants responded to brief questionnaires before, in the middle of the experiment (i.e., two weeks later), and after the experiment (i.e., four weeks later), including PHQ9, ISI, and GAD7. The Morningness Eveningness Questionnaire (MEQ) was used to measure the morningness or eveningness of the participants.

*sensor_hrv.csv* contains sensor and HRV measurements in 5-minute intervals, excluding periods when the device was off the wrist, no data was collected, or the signal was too noisy for reliable HRV extraction. The sensor information was aggregated using the mean value. Each row represents recordings for a 5-minute segment of a given user, as indicated by the *deviceId* column. Time is recorded in milliseconds using the epoch format. Each segment is defined by *ts_start* (start time) and *ts_end* (end time), with all other features computed within this interval. Each row additionally has a *missingness_score* column, which measures the level of error allowance in HRV analysis^27^ by quantifying the proportion of data within a segment that is affected by signal loss or artifacts during HRV analysis. This score reflects the degree of data unreliability: higher values indicate greater missing or invalid RR intervals. We collected a total of 33,600 hours of data from the participants, averaging 672 hours per person.

1. Sensor data. Our data spans the following sensor types as introduced in Table 2: (1) heart rate monitor along with interbeat interval (ibi), (2) accelerometer having X, Y, and Z components, (3) gyroscope having X, Y, and Z components, (4) gyroscope rotation vector having X, Y, and Z components along with W angle of rotation, (5) pedometer recordings, including number of steps, distance, and calories burned, and (6) light sensor.
2. HRV measurements. We computed the HRV features in Table 2 for each 5-minute segment from the PPG data stream. Heart rate and HRV recordings were computed by dividing continuous PPG signals from the smartwatch into 5-minute chunks using the HeartPy library in Python (https://python-heart-rate-analysis-toolkit.readthedocs.io). This open-source toolkit implements standard HRV computation methods following established guidelines, performing automated peak detection, artifact removal, and computation of both time-domain and frequency-domain features, computed from the time-domain features using the Fast Fourier Transform. As a consequence, SDNN, SDSD, RMSSD, PNN20, and PNN50 are categorized as time-domain HRV features; LF, HF, and LF/HF are the frequency-domain HRV features.

*sensor_hrv_filtered.csv* contains the same information as *sensor_hrv.csv*, with rows (5-minute segments). Data were filtered using a missingness score of 0.35 as a quality threshold, as suggested by previous research^24^.

*sleep_diary.csv* includes self-recorded participant sleep schedules from daily surveys. Users are distinguished by *userId*, and *date* represents the day participants filled out the survey. The participants provided the following estimates: go-to-bed time, fall-asleep time, wake-up time, and the duration of mid-sleep awake time, also called WASO (if applicable). Additionally, we enriched this information with commonly used quantifiable sleep metrics, such as sleep duration, in-bed duration, sleep efficiency, and sleep latency.

*raw_data/ppg.csv.gz* contains the raw PPG signal collected from the smartwatch. It includes the timestamp in epoch format, PPG signal, and device ID.

*raw_data/hrm.csv.gz* contains the heart rate monitor recorded by the smartwatch. It includes the timestamp in epoch format, heart rate, and device ID.

*raw_data/acc.csv.gz* contains the raw accelerometer recordings collected from the smartwatch. It includes the timestamp in epoch format, X-, Y-, and Z components of the accelerometer, and device ID.

*raw_data/grv.csv.gz* contains the raw gyroscope rotational vector recordings collected from the smartwatch. It includes the timestamp in epoch format, X-, Y-, and Z components, W angle of rotation of the gyroscope rotational vector, and device ID.

*raw_data/gyr.csv.gz* contains the raw gyroscope recordings collected from the smartwatch. It includes the timestamp in the epoch format, X-, Y-, and Z components of the gyroscope, and device ID.

*raw_data/lit.csv.gz* contains the ambient light brightness recorded by the smartwatch. It includes the timestamp in epoch format, ambient light brightness level, and device ID.

*raw_data/ped.csv.gz* contains the pedometer recordings collected from the smartwatch. It includes the timestamp in epoch format, number of steps, distance, burned calories, and device ID.

*README* provides a detailed explanation for all the fields in the presented files.

## Technical Validation

### High-level characteristics

**Fig. 4:**
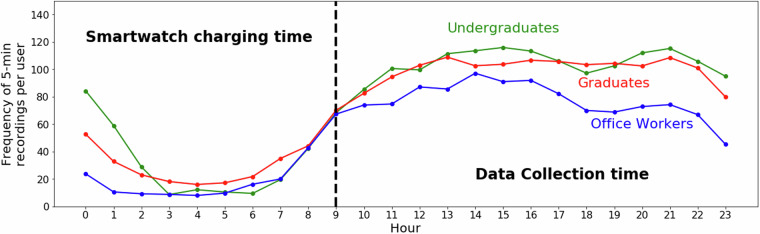
Average cumulative number of 5-minute smartwatch recordings per user across 24 hours, stratified by participant occupation (undergraduates, graduates, and office workers). The dashed vertical line at 09:00 separates typical smartwatch charging hours (00:00–09:00) from active data collection periods (09:00–23:00), which is the focus of all subsequent analyses.

To validate our data, we present overall observed trends using different approaches to assess its robustness. First, we show the frequency of 5-minute data points available in our dataset in Fig. 4. Although participants were only asked to wear the smartwatch during the day, we still observed some data collected during the night. While we have retained the night-time data in our dataset, we focus on daytime data for validation purposes. The plot shows that the highest frequency of 5-minute recordings per user is generally observed between 09:00 and 23:00. Thus, we limit our discussions in this paper to this period.

**Fig. 5:**
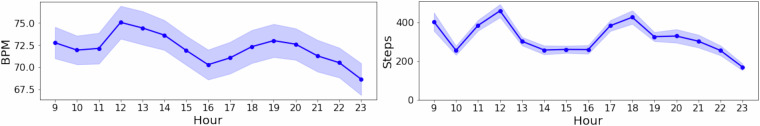
Aggregated hourly trends of heart rate (left; in beats per minute, BPM) and physical activity (right; steps per hour) across all participants between 09:00 and 23:00. Shaded regions represent standard errors around the mean values, highlighting periods of increased activity corresponding roughly to midday and early evening hours, typical times for meals and commuting.

**Fig. 6:**
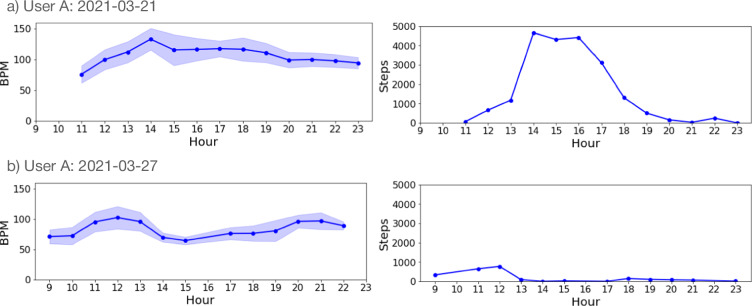
Representative daily recordings of heart rate (left; beats per minute, BPM) and physical activity (right; steps per hour) for a single participant (User A) on two different days (a: 21st March and b: 27th March). Shaded regions indicate the standard deviation of heart rate. The activity profile clearly illustrates distinct patterns that reflect typical intra-individual daily variability, such as a highly active (2021-03-21) *vs*. sedentary (2021-03-27).

Secondly, we present the movement data collected from smartwatches in Fig. 5. We observed that participants tend to take more steps during 11:00–13:00 and 17:00–19:00, which coincide with lunch and dinner times, respectively. This is an expected pattern for most students and office workers. Another way to validate our dataset is by analyzing the fluctuation patterns in heart rate (HR) values. We observed that HR follows a similar trend, with increased ranges during lunch and dinner times. The meal-related activity peaks reflect natural behavioral patterns, including meal preparation, which have been shown to contribute to increased daily step counts and overall physical activity^28^. Additionally, Fig. 6 shows an example of the same user recording on two different days. The user was active on 21st March with an HR ranging between 50–150 beats per minute (BPM) and more sedentary on 27th March with an HR ranging between 50–100 BPM.

**Fig. 7:**
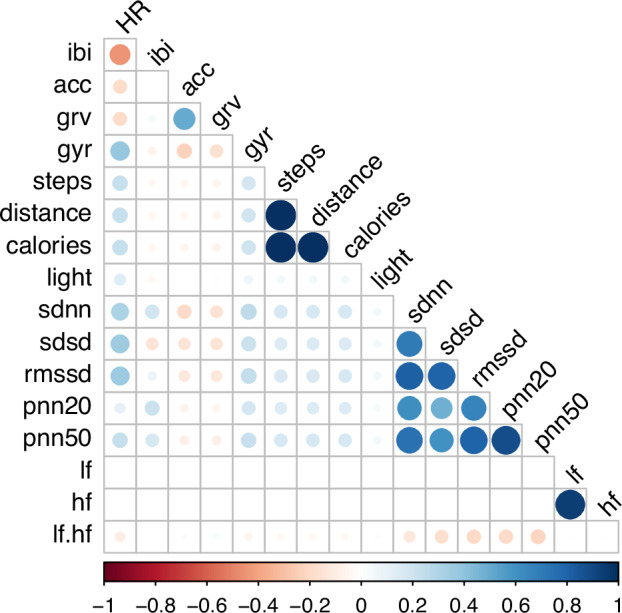
Correlation matrix of smartwatch sensor-derived signals and computed heart rate variability (HRV) features. Positive correlations are represented in blue, negative correlations in red, and circle size indicates correlation strength. The figure highlights interrelations among time-domain HRV measures (e.g., SDNN, SDSD, RMSSD, PNN20, PNN50) and their associations with activity (accelerometer, steps) and physiological measures (heart rate).

We also validated our dataset by presenting repeated-measures correlations^29^ between collected sensor recordings and computed HRV features (Fig. 7). This method assesses within-subject associations while accounting for inter-individual differences. For the accelerometer, gyroscope, and gyroscope rotational vector, we computed the overall magnitude by combining the x, y, and z components using the Euclidean norm. Given that smartwatch and HRV data were continuously collected throughout the day for each participant, while sleep diaries and clinical questionnaires were collected much less frequently (daily and biweekly, respectively), we omit them from this correlation.

We observed high positive correlations among all time-domain HRV features. Additionally, the frequency domain LF/HF exhibits moderate negative correlations with time-domain features, specifically showing a negative correlation with SDSD, RMSSD, PNN20, and PNN50. Finally, time-domain HRV features exhibited positive correlations with heart rate, pedometer recordings, and gyroscope data.

### HRV characteristics

**Fig. 8:**
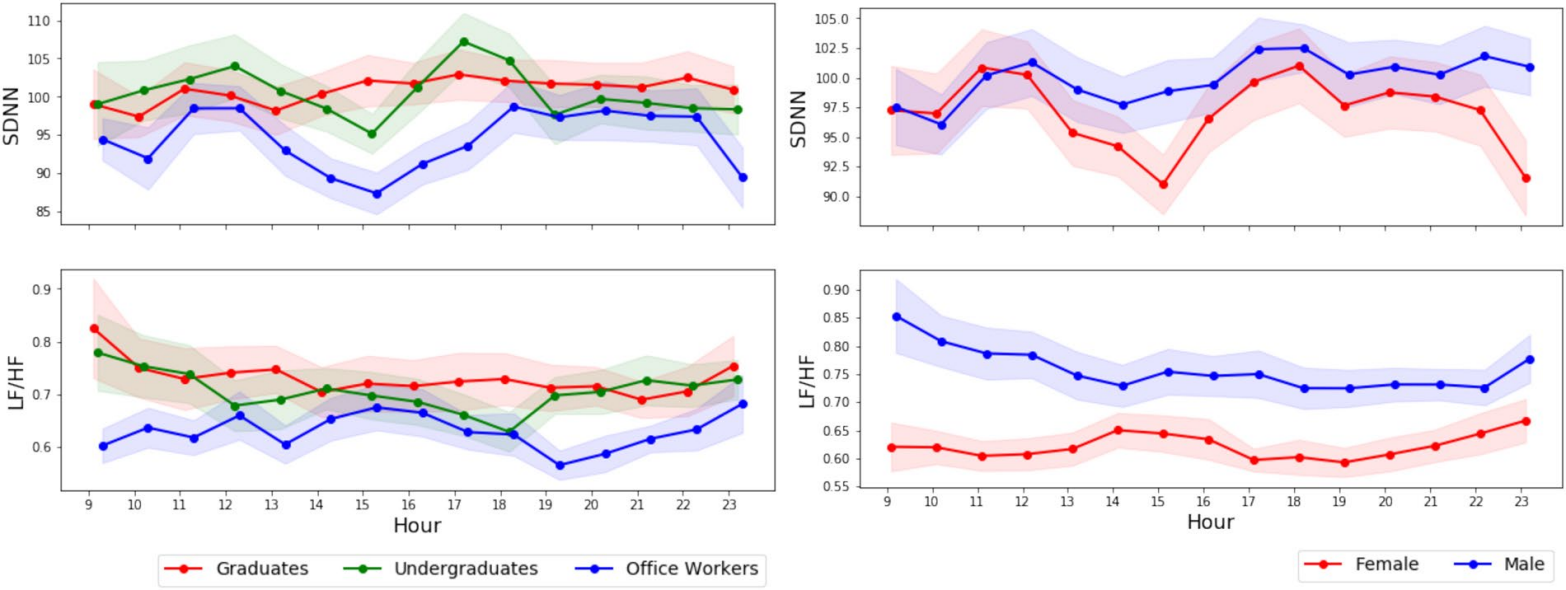
Hourly distribution of HRV features—SDNN (time domain, top) and LF/HF ratio (frequency domain, bottom)—aggregated by participant groups based on occupation (left: undergraduates, graduates, office workers) and gender (right: female, male). Shaded regions represent standard errors around the hourly means.

The distributions of HRV features over one day and one week by occupation group (undergraduates, graduates, and office workers) are shown in Fig. 8. As all time-domain indicators, including RMSSD, SDNN, SDSD, PNN20, and PNN50, are highly correlated, we only present detailed statistics for one time-domain feature, namely SDNN. It was chosen as a more robust feature, being less sensitive to isolated artifacts due to its reliance on overall variability across a segment^30^. SDNN ranges between 85 ms and 110 ms per user and appears to be higher in the group of undergraduate students. For the frequency-domain feature, namely the LF/HF ratio, we observe a fluctuation between 0.5 and 0.9. The trend that undergraduate students have higher SDNN values than office workers may align with the previous findings that HRV features decrease with age^31^. Moreover, the trend of female participants exhibiting lower HRV measurement values aligns with the results of a large-scale meta-analysis showing that women generally exhibit reduced overall HRV as reflected in lower LF/HF and SDNN^32^. The overall trend of HRV features within a day, such as a gradual decrease of LF/HF in male participants and lowering of SDNN in the early afternoon period, further suggests the circadian rhythms of HRV variables, which validates previous research findings^33–35^.

To validate our computed HRV ranges, we verified the ranges of HRV measures with existing standards. The descriptive statistics of our data are reported in Table 3. We aggregated data per user before obtaining the final values presented in the table, i.e., the mean of means. We used the mean of means approach for the following three reasons specific to our dataset structure: (1) participants contributed varying amounts of valid data due to different compliance levels and off-wrist periods. Current approaches to longitudinal data analysis emphasize that unbalanced designs require methods that prevent disproportionate weighting of individuals with more complete data^36^; (2) this approach also aligns with established HRV assessment practices, where individual baseline characteristics are emphasized for clinical interpretation^37^; (3) we acknowledge that this approach reduces apparent variability compared to raw data pooling. However, it provides more representative population estimates while ensuring each participant contributes equally to the overall data analysis regardless of their total valid measurements, which is fundamental to obtaining unbiased group-level estimates in longitudinal studies with missing data.

**Table 3:** Descriptive statistics of computed short-term HRV features.

HRV Features	Mean	STD	Min	Max
SDNN (ms)	98.7	9.9	76.7	119.1
SDSD (ms)	71.6	8.0	53.3	90.0
RMSSD (ms)	108.2	13.4	82.8	140.8
PNN20 (ms)	0.76	0.04	0.64	0.85
PNN50 (ms)	0.48	0.07	0.35	0.66
LF (ms^2^)	1549	390	737	2645
HF (ms^2^)	3299	729	1809	5017
LF/HF	0.69	0.15	0.43	1.10

Mean values, standard deviations, and ranges (minimum and maximum) for time-domain features (SDNN, SDSD, RMSSD, PNN20, PNN50) and frequency-domain features (low frequency [LF], high frequency [HF], and LF/HF ratio) calculated from 5-minute HRV segments are presented.

We observed that the SDNN intervals in our data align with those reported in previous studies^31,38^. Similarly, a previous study measuring the LF/HF ratio over a month-long time in desk workers reported that the average values ranged between 0.54 and 0.59^39^. Although the measurement procedure in our experimental setup differs significantly from that in the previous study, which used a camera-sensing system, it is noteworthy that our intervals generally align with their data.

There is difficulty in directly comparing the current data with prior work due to the unique *in-situ* context of our experiment. Additionally, HRV values are context-dependent, varying considerably with gender and age^40^. Unlike most published research, which gathers data in a controlled clinical environment during resting states, we collected recordings 24/7 using wearable smartwatch devices, which inevitably contain higher noise levels that arise in real-life, uncontrolled environments. In particular, we observed that RMSSD is different in short-term measurements in the existing literature, possibly due to its higher sensitivity to artifacts (i.e., incorrectly detected peaks)^30^. Nonetheless, the PPG-derived RMSSD values were generally reported not to exceed 130 ms (https://python-heart-rate-analysis-toolkit.readthedocs.io/en/latest/heartrateanalysis.html), which is confirmed by our mean value variation of 108.2 ± 13.4 ms. We note that there is a lack of literature on establishing standard values for continuous HRV features. Another crucial distinction is that while we derive HRV from PPG signals, other studies reporting these ranges typically use ECG signals^14^, further complicating the comparison.

### Sleep diary characteristics

To validate our sleep diary data, apart from ensuring participants kept a robust daily record, we also compared our data with published studies. Table 4 compares key sleep metrics of our diaries with other datasets. We observe that the self-reported bedtime presented in our data tends to be earlier than the time in the other dataset, which used smart bands for collecting data^41^. These findings are consistent with the previous work analyzing sleep patterns among multiple countries and data collection approaches^42,43^.

**Table 4:** Descriptive statistics of data collected through the sleep diaries.

Sleep indicator	All	Office workers	Graduates	Undergraduates	Smart bands (students)^40^
WASO (min)	15 ± 19	17 ± 15	21 ± 28	5 ± 4	54 ± 26
Sleep duration	7 h 49 min (49)	7 h 55 min (48)	8 h 5 min (47)	7 h 22 min (46)	6 h 9 min (112)
In-bed duration	8 h 23 min (54)	8 h 26 min (44)	8 h 45 min (47)	7 h 51 min (62)	7 h 3 min (127)
Sleep latency (min)	34 ± 25	31 ± 21	40 ± 31	29 ± 23	54
Sleep efficiency	0.94 ± 0.04	0.94 ± 0.04	0.93 ± 0.05	0.94 ± 0.04	0.87 ± 0.04
go-to-bed	00:38	23:40	00:48	01:42	02:46
wake-up	09:01	08:06	09:33	09:33	09:48

Mean values (with standard deviations in parentheses or indicated after ±) for sleep indicators, including wake after sleep onset (WASO), sleep duration, in-bed duration, sleep latency, sleep efficiency, and bedtime/wake-up times, are provided. Metrics are stratified by participant groups (office workers, graduates, undergraduates) and compared against data from an existing study on student populations using smart bands.

### User-level validation

Since the dataset validation presented above is based on aggregated trends, we next validated recordings at the individual user level. We confirmed that the sensor values from the smartwatch fall within the acceptable ranges specified in the device manual. Specifically, we verified that the proportion of collected PPG signals within the valid range (0 to 4, 194,304) lay between 85.6% and 99.9% across all users. Although this proportion is high for most users, we still observed instances where PPG signals fall outside this range, which may indicate periods when the smartwatch was off the wrist. For HRV computations, these intervals were discarded; however, we recommend carefully identifying and removing artifacts in the raw PPG signal for further analysis.

## Usage Notes

### Potential applications

We present data extracted from wearable devices over four weeks, licensed under a Creative Commons Attribution 4.0 International License. This dataset can be used to develop various applications, including predictive analytics, for example, for important psychological indicators.

More specifically, our continuous wearable data, combined with clinical symptom questionnaires, will be valuable for predicting the severity of common mental health conditions such as insomnia, which is characterized by difficulty falling and staying asleep. For example, all collected features could be used as independent variables to predict physiological conditions associated with the Insomnia Severity Index (ISI). Similarly, researchers could assess the impact of sensor data, including pedometer readings and gyroscope rotational vectors (GRV). While this represents an initial step in exploiting data collected by wearable devices for predicting physiological well-being, these findings may highlight the significance of HRV features in this task.

Other potential applications extend to predictive analysis using deep-learning-based algorithms and representation learning with multimodal data such as HRV and activity features. We hope that the dataset presented in this work will serve as a benchmark for research into well-being using AI predictive analytics. This dataset enables researchers to use continuous physiological signals retrieved from mobile wearable devices to predict and understand daily human mental health indicators.

Moreover, this dataset can facilitate sleep research through group-level analyses. For example, by applying unsupervised clustering on wearable data, unique traits for groups with the most irregular sleep patterns can be determined^44^. Distinguishing groups—by exercise intensity, lifestyle regularity, or bedtime and wake-up times—can reveal differences in HRV and sleep patterns^33^. Propensity score matching analysis could further explore how individual efforts affect well-being. Researchers can also treat our dataset as conventional short-term (5-minute) HRV data by selecting specific periods each day.

### Limitations

#### Data collection medium

Smartwatch-based PPG sensors are susceptible to noise due to the different styles of wearing the watch. It is also relatively easy to remove the watch during the experiment, leading to off-wrist periods across participants. In such cases, we rigorously pre-processed the data to remove possible noise, resulting in missing data for some periods. Further data imputation may be necessary. Additionally, this dataset focuses on daytime recordings as participants were instructed to charge their watches overnight. Regarding sleep diaries, participants relied on their memory when answering the questionnaires, potentially introducing perceptual bias.

#### Demographics

The dataset is restricted by not representing the overall population in terms of occupation, age, and country of residence.

#### Possible bias in HRV computation

We used HeartPy (version 1.2.5), a Python open-source library specifically developed for analyzing noisy photoplethysmography (PPG) data collected from wearable devices^45^. HeartPy was selected because most available heart rate analysis algorithms are designed for electrocardiogram (ECG) data, which have different signal properties compared to PPG signals from consumer wearables. The library employs an adaptive threshold-based peak detection algorithm with moving average filtering to accommodate morphology and amplitude variations in PPG waveforms. The superior performance of this approach in comparison to other open-source algorithms was validated on noisy PPG data^45^. However, the library did not successfully detect peaks in some 5-minute segments due to noise or insufficient signal quality, resulting in missing HRV values for these periods. If researchers choose different HRV computation methods or libraries (such as pyPPG (https://pyppg.readthedocs.io) and NeuroKit2 (https://neuropsychology.github.io/NeuroKit)), they may obtain different results due to variations in preprocessing, peak detection algorithms, and parameter settings, as different methods handle noisy signals differently^45^.

## Data Availability

The Python code for extracting HRV features from the raw PPG signal can be accessed at https://github.com/aitolkyn99/hrv_smartwatch. The Python Heartpy package was utilized to compute HRV measurements. For further information, dataset users can contact the authors.
